# Sequencing of IncX-Plasmids Suggests Ubiquity of Mobile Forms of a Biofilm-Promoting Gene Cassette Recruited from *Klebsiella pneumoniae*

**DOI:** 10.1371/journal.pone.0041259

**Published:** 2012-07-23

**Authors:** Mette Burmølle, Anders Norman, Søren J. Sørensen, Lars Hestbjerg Hansen

**Affiliations:** Department of Biology, University of Copenhagen, Copenhagen, Denmark; Aarhus University, Denmark

## Abstract

Plasmids are a highly effective means with which genetic traits that influence human health, such as virulence and antibiotic resistance, are disseminated through bacterial populations. The IncX-family is a hitherto sparsely populated group of plasmids that are able to thrive within *Enterobacteriaceae*. In this study, a replicon-centric screening method was used to locate strains from wastewater sludge containing plasmids belonging to the IncX-family. A transposon aided plasmid capture method was then employed to transport IncX-plasmids from their original hosts (and co-hosted plasmids) into a laboratory strain (*Escherichia coli* Genehogs®) for further study. The nucleotide sequences of the three newly isolated IncX-plasmids (pLN126_33, pMO17_54, pMO440_54) and the hitherto un-sequenced type-plasmid R485 revealed a remarkable occurrence of whole or partial gene cassettes that promote biofilm-formation in *Klebsiella pneumonia* or *E. coli*, in all four instances. Two of the plasmids (R485 and pLN126_33) were shown to directly induce biofilm formation in a crystal violet retention assay in *E. coli*. Sequence comparison revealed that all plasmid-borne forms of the type 3 fimbriae encoding gene cassette *mrkABCDF* were variations of a composite transposon Tn*6011* first described in the *E. coli* IncX plasmid pOLA52. In conclusion, IncX-plasmids isolated from *Enterobacteriaceae* over almost 40 years and on three different continents have all been shown to carry a type 3 fimbriae gene cassette *mrkABCDF* stemming from pathogenic *K. pneumoniae*. Apart from contributing general knowledge about IncX-plasmids, this study also suggests an apparent ubiquity of a mobile form of an important virulence factor and is an illuminating example of the recruitment, evolution and dissemination of genetic traits through plasmid-mediated horizontal gene transfer.

## Introduction

Plasmids are autonomously replicating extra-chromosomal elements that are ubiquitous within most studied natural microbial communities and clinical pathogens. Apart from encoding specific ‘selfish’ traits that ensure continuing and stable propagation, most studied plasmids have been found to carry genetic payloads that provide certain adaptive advantages, in effect compensating for the metabolic burden they impose upon the host [1]. As a consequence, plasmids are often found to contribute significantly to the accretion and dissemination of genes providing hosts with clinically relevant traits in the form of antimicrobial resistance or virulence, which potentially facilitate or aggravate infections. Accordingly, several of the members of the family *Enterobacteriaceae* such as *Klebsiella*, *Proteus* and uropathogenic strains of *Escherichia coli* (UPEC), are able to produce surface structures (fimbriae) that promote attachment to epithelial cells as well as adhesion to many types of abiotic surfaces [2]. In particular, they cause problems with keeping abiotic surfaces sterile in hospital settings, leading to conditions such as urinary tract infections (UTI) [3]. As surface structures like fimbriae and conjugal pili are essential in the establishment of these kinds of bacterial plaques or biofilms, the occurrence of biofilm promoting genes on mobile genetic elements [4], [5] represent an alarming development in need of close monitoring.

In order to appreciate the extent with which genes such as these are spread throughout bacterial communities, a good understanding of the basic genetic framework that governs the dynamics of gene accretion and dissemination is vital [1]. The type of plasmid that carries such adaptive genes can for example greatly determine the range of bacterial hosts to which these potentially harmful genes can be spread.

Plasmids have traditionally been divided into discrete groups (families) based on the inability of closely related plasmids to propagate stably within the same bacterial strain [6], [7], which is why these groups are commonly referred to as incompatibility (*inc*) groups (e.g. IncP, IncI, IncF, IncX, IncN, IncW etc.). The IncX group is a relatively poorly studied group of plasmids that has mostly been associated with members of the family *Enterobacteriaceae* residing within the guts of animals [8], [9]. This group, however, had until recently not received much attention with respect to the kinds of adaptive loads they can carry, but genetic analysis of the IncX plasmid pOLA52 (GenBank accession number NC_010378.1), revealed a number of surprising traits that were in all likelihood recruited directly from the chromosome of *Klebsiella pneumoniae*. These included the two composite transposons Tn*6010* and Tn*6011* which confer wide-spectrum multidrug resistance through a large resistance nodulation-division (RND) type efflux pump and type 3 fimbriae-mediated biofilm formation, respectively [10]–[12].

In an effort to locate and characterize more plasmids from the IncX group without specific regard to the carriage of genetic payloads, we applied a replicon-centric screening method to a large number of wastewater isolates, which resulted in the capture of three novel IncX-plasmids.

All captured IncX-plasmids were genome sequenced and analysed. The IncX group has so far only been characterized as containing two distinguishable subgroups, IncX1 and IncX2 represented by the plasmids R485 and R6K, respectively. The IncX2 prototype R6K was sequenced at the Sanger Institute in 2006 (sequence in un-annotated form is available at http://www.sanger.ac.uk/Projects/Plasmids/), while the R485 plasmid was sequenced in this study.

The plasmids investigated in this study were shown all to contain genes related to biofilm promotion. These results indicate that a specific biological link exists between biofilm promoting gene cassettes and the IncX group of plasmids.

## Materials and Methods

### Bacterial Strains and Growth Conditions

All strains of *Enterobacteriaceae* (including *E. coli* GeneHogs®) were grown at 37°C in LB broth, MacConkey agar (DIFCO) without supplements or on LB agar supplemented with antimicrobial agents in the following concentrations: Ampicilin (Ap) 70 µg ml^−1^, Kanamycin (Km) 50 µg ml^−1^, Streptomycin (Sm) 100 µg ml^−1^.

### Screening of Wastewater MacConkey Isolates for Plasmids with IncX-type Replicons

Wastewater was sampled from two Danish water treatment plants (WWTPs). One sample (Mølleåværket, Lyngby DK - 55°48′8′′N, 12°32′23′′E) was taken from the primary sedimentation tank of the WWTP, while another was taken from the activated sludge basin (Lynetten, Copenhagen DK - 55°41′44′′N, 12°36′59′′E).

From each wastewater sample 1 mL was diluted in phosphate buffered saline (PBS) to a factor of 10^6^ and plated out on MacConkey agar, which was then incubated for 24 h. A total of 960 individual colonies (480 from each of the samples) were subsequently replica plated on LB agar without supplements, and LB agar supplemented with streptomycin or ampicillin respectively. Freeze cultures were also made from these colonies with glycerol as a cryoprotectant that were subsequently kept frozen at −80°C.

A library of total genomic DNA was created by the following method: Single colonies were picked and pooled together 8 at the time in 100 µL PBS and mixed by vortexing for 30 seconds. Pooled cell samples were washed with an equal volume of PBS, and subsequently resuspended in equal volumes of PCR grade ddH_2_O. Sample lysates were made by transferring 10 µL aliquots to a PCR multiwell plate and subjected to a PCR cycle consisting of 10 minutes at 95°C. Lysates were subsequently frozen and kept at −18°C until needed.

Lysates were screened for IncX-type replicons with multiplex PCR, using primers specific for IncX1- (Forward: 5′-AACAGCACATTCTCATCGTT-3′, reverse: 5′-CTCTTCTTGCGACTCTCTCA) and IncX2-replicons (Forward: 5′-CGAAACGAGCTAAATCACAC-3′, reverse: 5′-AGTGAAGCCAACAAACGATC-3′), and visualized with agarose gel electrophoresis. Positive bands led to a second round of screening of individual candidates within that pool using colony PCR, but with the two primer-sets run in separate PCR reactions.

### Transposon Aided Plasmid Capture (TAPC) of IncX-plasmids

Total plasmid DNA isolations of IncX positive strains were performed using the Plasmid Mini AX kit (A&A Biotechnology, Gdynia, Poland), and subsequently visualized on agarose gels to assess the plasmid composition. To isolate plasmids carrying IncX replicons from any other resident plasmids, the transposon Entranceposon-KanR, which carries a Kanamycin resistance cassette, was inserted randomly by adding it to a small volume of plasmid eluate together with MuA-transposase (Finnzymes), according to the manufacturer’s instructions. Transposase/DNA mixtures were then transformed into electrocompetent *E. coli* GeneHogs® (Invitrogen, Carlsbad, CA) followed by selection on LB agar supplemented with kanamycin. IncX-positive transformants were located with colony PCR using the relevant IncX primer-sets specified above. These colonies were subsequently subjected to plasmid purifications and the presence of only a single plasmid in each transformant was verified through visualization on agarose gels.

### Sequencing

All plasmids were sequenced on the GS sequencer FLX high throughput platform (454 Life Sciences, Branford, CT, USA), using a procedure slightly modified from the one outlined by the manufacturer.

Plasmid templates were prepared from Plasmid Mini AX Kit purifications. In cases where the total plasmid yield was less than 5 µg, the plasmid preparations were amplified with multiple displacement amplification using the Repli-g kit (Qiagen, valencia, CA) to ensure adequate amounts of template. Five µg of DNA was used to construct single stranded (ssDNA) libraries according to the procedure outlined in the GS FLX Library preparation manual (Roche). The ssDNA libraries were quantified with qPCR using primers targeting the A & B adaptors, a 180 bp ssDNA standard (a kind gift from Tom Gilbert) and the Brilliant SYBR Green QPCR Master mix (Stratagene, Cedar Creek, TX). A series of emulsion PCR reactions (emPCR) were performed in order to reach an optimal copy/bead ratio. DNA carrying beads were subsequently sequenced using the LR25 Sequencing Kit on either one or two small regions.

16 S rRNA sequencing was done using the Sanger method and performed by Macrogen, Korea with 27 F and 1492 R primers [13].

### Genome Assembly and Finishing

Initial sequence assembly was performed with newbler 2.6 (454 Life Sciences, CT, USA), and subsequent sequence finishing was done using consed [14]. Chromosomally derived contigs were removed from each assembly prior to sequence finishing by calculating average base coverage for individual contigs (using a local Perl script) and removing those that were orders of magnitude lower than the mean coverage for contigs longer than 1000 bp long. Due to a high number of unresolved contigs, final verification of contig order and gap closing was confirmed with PCR and Sanger sequencing (Macrogen) in the case with the plasmids pMO17_54 and pMO440_54. The complete annotated nucleotide sequences for R485, pMO17_54 and pLN126_33 were uploaded to the European Nucleotide Archive (EMBL-Bank) and have been assigned the accession numbers HE577112, HE578057 & HE578058, respectively. Since the plasmids pMO17_54 and pMO440_54 were deemed to be identical, pMO440_54 was not uploaded to the database.

### Sequence Analysis

Putative coding regions were located with prodigal [15]. Overall gene annotations were performed in artemis. Nucleotide sequence and protein homology was performed with the blastp program [16] against the GenBank databases. Blastx searches were also used to look for small or disrupted coding sequences. Insertion sequences within the nucleotide sequence were located by using is finder (http://www-is.biotoul.fr/). The program tmhmm [17] was used for predicting transmembrane-helices in selected protein sequences.

### Biofilm Formation Assay

The ability of *Escherichia coli* Genehogs containing plasmids R485, pLN126_33, pMO17_54 and pMO440_54 to promote biofilm formation was based on quantification of attached cells by use of crystal violet (CV) as described previously [18]. Single colonies of each strain were transferred from LB plates to 5 ml of M9 minimal medium containing selective antimicrobial agents where required, and incubated at 37°C ON. Aliquots of 20 µl of ON culture of each strain were transferred to flat bottomed microtiter plate wells (in four replicates) containing 180 µl M9 minimal medium, and plates were closed with lids, sealed with parafilm and incubated at 37°C ON with agitation at 250 rpm. Staining and quantification of attached cells was performed as previously described [9], using an EL 340 microplate reader (Bio-Tek Instruments, Winooski, Vermont) for absorbance measurements at 595 nm. Absorbance measurements of wells containing only M9 minimal medium were used as a baseline.

## Results and Discussion

### Selection-independent Isolation of IncX-replicon Plasmids from WWTPs

Wastewater is an abundant source of gut microbes, including those belonging to the family *Enterobacteriaceae*. Nine hundred and sixty wastewater isolates able to grow on rich bile-salts medium (MacConkey) were screened for the presence of IncX-replicons using primers specific to the replication initiation gene *pir* of pOLA52 (the IncX1 group), and the well-studied *pir* gene from R6K (IncX2 group).

A total of 3 strains out of 960 were found to be IncX1-positive while no strains where found to be IncX2-positive. Although the sample size is relatively small, the ubiquity of IncX1 type plasmids, as opposed to IncX2, correlates well with at least 7 occurrences of identical homologues of the pOLA52 *pir* gene, but only one occurrence of the IncX2 type (from R6K itself) within the GenBank nucleotide database.

A preliminary screening for antibiotic resistance revealed that 80% of the WWTP isolates conferred resistance towards ampicillin and 29% conferred resistance towards streptomycin (only 4% conferred resistance towards both). None of the IncX1-positive strains, however, were shown to confer resistance towards either of these antimicrobial agents.

Sequencing of the 16 S rRNA regions of the three IncX1-positive strains and subsequent classification using the Ribosomal Database Project Naïve Bayesian Classifier [19] confirmed that all three strains came from within the *Enterobacteriaceae*. All IncX-positive isolates were assigned to the mixed-genus class *Escherichia*/*Shigella* with a posterior probability of 1.0 (i.e. 100% confidence). The phylogenetic distance to the *Shigella flexneri* reference sequence was shown to be infinitesimally smaller than to *E. coli* in all three instances (not shown). Thus, the three strains will in following be referred to as *Shigella* sp.

### Plasmid Carriage

**Figure 1:**
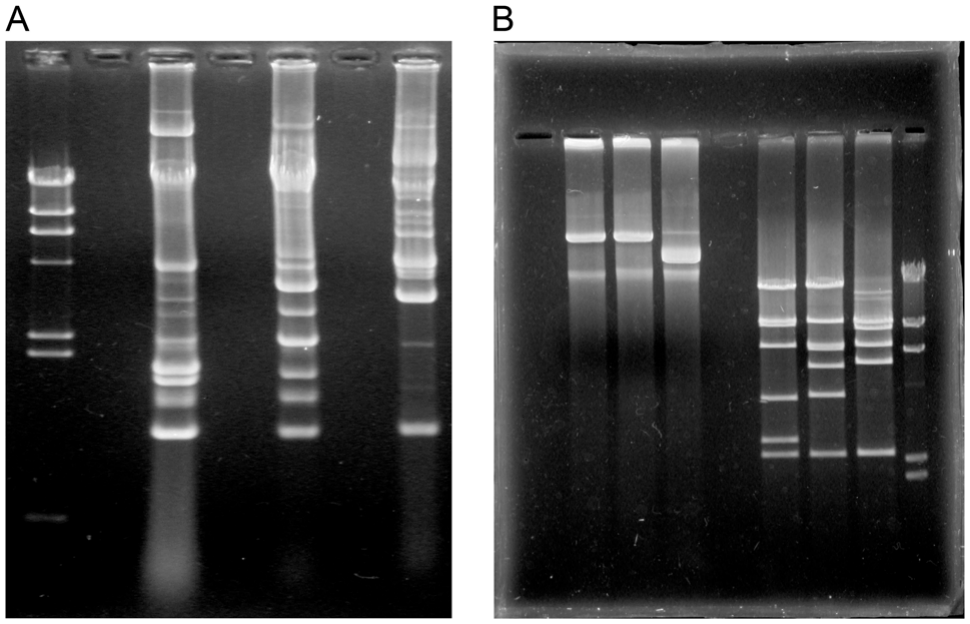
Capture of IncX-replicon plasmids. Agarose gel electrophoresis of (a) plasmid purification of strains *Shigella* sp. MO17, MO440 and LN126, respectively, followed by (b) plasmid purifications of IncX-replicon positive transformants of Entranceposon derivatives of the same strains. The leftmost three lanes are *Eco*RI digests of the three first lanes. Based on the sequence analysis, the plasmids of MO17 and MO440 were identified as being identical, only differing by the position of the Entranceposon. This difference in Entranceposon insertion site accounts for the dissimilar band pattern observed within the digested versions of these two plasmids (see text for more details).

Plasmid profiles of the three IncX-positive strains *Shigella* sp. LN126, *Shigella* sp. MO17 and *Shigella* sp. MO440, revealed a remarkable presence of extrachromosomal elements **(** **Fig. 1a** **)**. In addition to a recurring band normally consisting of sheared chromosomal DNA, each of the plasmid purifications contained between 15–20 bands, hinting at the presence of several different extra-chromosomal elements of various sizes in all three strains.

All three IncX1-replicon containing plasmids were successfully transferred from their host strains and other extra-chromosomal elements into *E. coli* GeneHogs®, by using the TAPC method described in the materials & methods section. Subsequent localization of IncX1-positive transformants was done through a progressive PCR screening procedure similar to the one used for screening of WWTP isolates (**Fig. 1b** **)**. Thirteen IncX1-positive Entranceposon derivatives out of 100 where found from the *Shigella* sp. MO17 transformants, while only 1 in 500 and 1 in 620, were found from the *Shigella* sp. MO440 and LN126 transformants, respectively. Thus, it appears that the overall plasmid carriage can dramatically affect the outcome of this procedure. A described below, the two plasmids pMO17_54 and pMO440_54 were revealed to be identical, and the large difference in the occurrence of the IncX-positive Entranceposon derivatives is therefore remarkable. Since each replicon within a strain is a potential recipient of the Entranceposon, a high occurrence of small high copy number plasmids will most likely come to dominate the fraction of Entranceposon derived transformants. If one specifically aims at capturing larger plasmids with TAPC, we suggest including measures to reduce the number of small high copy number plasmids before random Entranceposon insertions are performed.

Nevertheless, TAPC screening seems to be a viable technique for capturing specific plasmids from culturable environmental isolates that contain numerous extra-chromosomal elements, although the approach still has room for improvement.

### Sequence Analysis of Captured IncX-replicon Plasmids and R485

**Figure 2:**
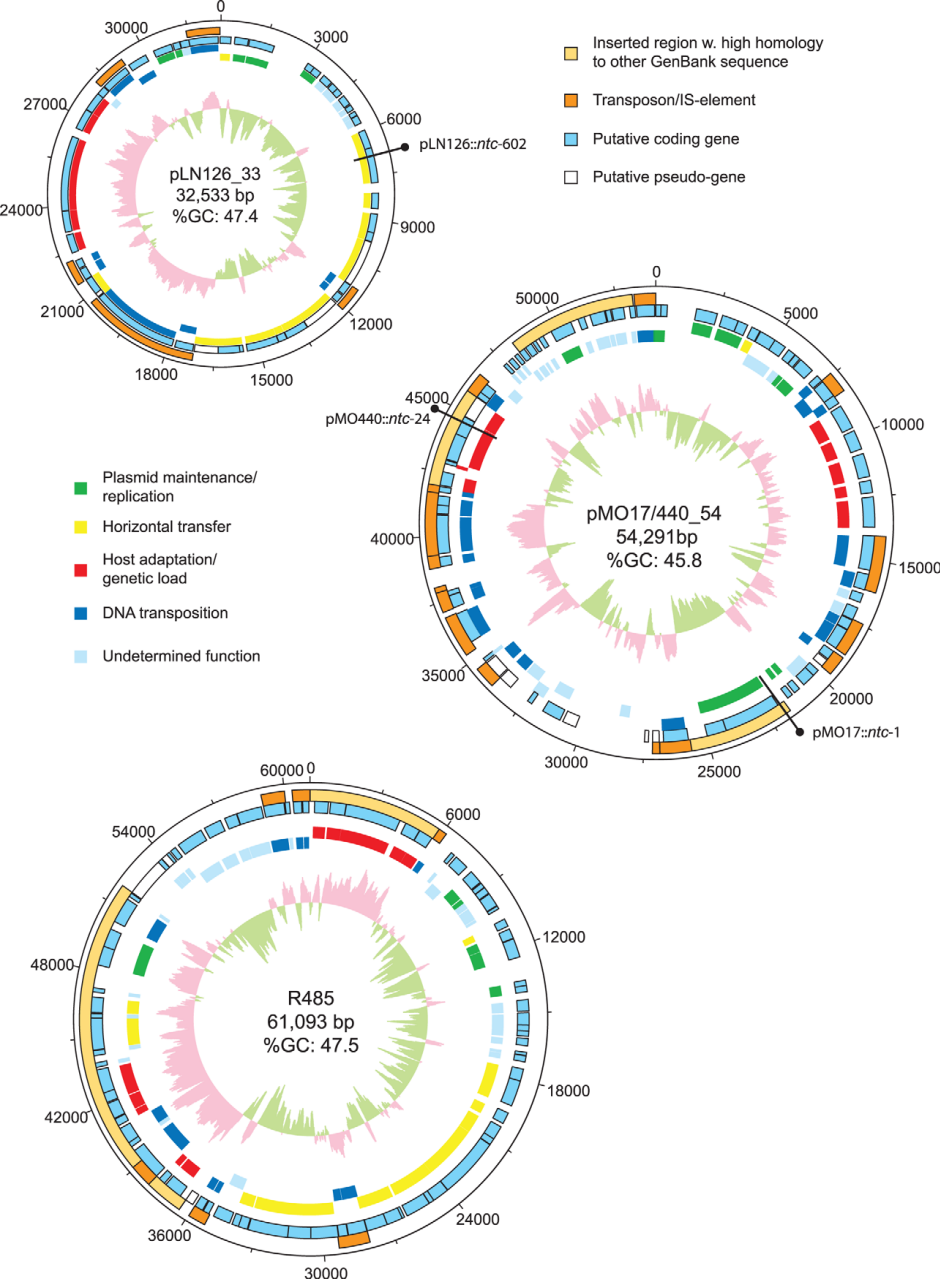
Sequence Maps. Graphical representation of the annotated IncX-type plasmids, pLN126_33, pMO17/440_54 and R485. The central diagrams represent the %GC content. Black pins represent the insertion points of the Entranceposon derivatives.

The four sequences consisted of circular plasmids of 54, 54, 33 (thus, pMO17_54, pMO440_54 and pLN126_33), and 61 kilobases (kb) in size, respectively, and were therefore within the normal size-range reported for IncX-plasmids (30 kb –80 kb). The inserted Entranceposon was excised from the finished sequences of pLN126_33, pMO17_54 and pMO440_54. The base composition of the plasmids were all in the 45–50%GC-content range, consistent with most reported *Enterobacteriaceae* genomes [20]. Comparison of the sequences of pMO17_54 and pMO440_54 revealed enough nucleotide similarity to conclude that they were identical (except for the inserted Entranceposon which was removed from the finished sequence). Therefore, in the following sections, these two plasmids will be referred to collectively as pMO17/440_54 unless otherwise stated. A graphical representation of the sequenced plasmids from this study is presented in **Fig. 2** **.**

### pMO17/440_54

Annotation revealed a total of 71 putative protein-coding regions. The plasmid appeared highly mosaic in nature, with almost 27% of the plasmid being made up of insertion sequences (IS), transposons or fragments thereof. Furthermore, several regions displayed dramatic fluctuations in GC content, which also points towards a sequence of a highly composite nature.

#### Plasmid maintenance regions

About 22% of the plasmid consisted of two regions with high homology to maintenance regions (backbones) of the IncX-plasmid pSE34 (0–7.4 kb) and the IncFII-plasmid pO26-L (48.3–53.2 kb), respectively. The IncX-backbone region contained proteins related to the familiar PI encoding gene *pir* [4], (which in the following will be named *repX* so as to be more consistent with the naming of most other plasmid replicases), stability determinants in the form of a *parFG* partitioning locus characterized in the plasmid TP228 [21], and the *stbDE* plasmid addiction locus, characterized previously in R485 [22]. The upstream region of the *repX* gene, which is normally dedicated to replication initiation control in the form of iteron repeats [23], showed that a 77 bp deletion had reduced the normal number of five iterons to just one, and thus represented a substantially truncated replicon. Furthermore, the plasmid sequence did not show obvious signs of the secondary or tertiary replication origins that are normally associated with plasmids of the IncX group [24]. The replication initiation gene *repX* was not immediately followed by the *bis* and *ddp3* genes, as in most other described IncX-plasmids [4], [5], [24]. Instead, *repX* was followed by a ∼300 bp intergenic region and a gene (*repX2*) which also encoded a putative replication initiation protein. A homology search revealed that the second protein was a divergent homologue (47% similarity) of the PI protein of IncX-plasmid p2ESCUM, which shows slightly closer similarity to the IncX2 replicon of the *E. coli* plasmid R6K. Rather curiously, the whole IncX-backbone region was surrounded by IS26-elements, meaning that this backbone region in effect could constitute a small mobile element in the form of a composite transposon.

The IncFII-replication region only contained a single protein-encoding gene with an identifiable function. This was the antisense-RNA controlled replication initiation gene *repFII* characterized in plasmids like R1 [25].

A region (27.3–30.8 kb) contained a pseudo-gene with significant (E = 2e-65) blastx homology to a replication initiation protein from the *E. coli* plasmid pMG828-2 (GenBank ID: DQ995352), making it likely that this region previously functioned as a plasmid replicon. The replicon mosaic-like structure observed in the pMO17/440_54 plasmid may indicate that some plasmids, over time, can host several different replicons depending on the flow of foreign insertions, recombinations, and various intra- or extracellular selective pressures.

#### Horizontal transfer

Uncharacteristically for such a large plasmid [26], pMO17/440_54 did not contain any identifiable genes directly related to horizontal dissemination. The absence of genes encoding anything resembling mobilization relaxases or type IV secretion systems [27] would indicate that the plasmid is neither conjugative nor mobilizable. This was consistent with the fact that mating experiments failed to produce any transconjugants with the Entranceposon derivatives, although this could also have been caused by the disruption of vital transfer functions by the Entranceposon itself as in the case with pLN126_33 (see below). The presence of the hypothesized mobilization auxiliary gene *ddp3* gene on the plasmid and the cognate long inverted repeat containing an origin of transfer (*oriT*), however, could indicate that pMO17/440_54 might be indirectly mobilized by the presence of the TaxC relaxase encoded by another plasmid. A similar phenomenon can also be observed in the IncX-replicating *E. coli* plasmid pMccC7-H22 [28], which lacks an identifiable relaxase, but also still contains the replicon associated *oriT*.

#### Genetic load regions

The parts of pMO17/440_54 predicted to provide the host with adaptive traits, consisted of two inserted regions surrounded by IS-elements. One was a region (8.5–14.0 kb) in which a possible drug resistance mechanism was located, and the other (42.1–45.9 kb) encoded proteins associated with biogenesis of type 1 fimbriae.

The region containing a putative resistance mechanism did not display close nucleotide homology to any known genomic sequence. This region was however found to encode an AraC-like transcription regulator, two putative catabolic proteins and two putative membrane-spanning components. One of the putative enzyme encoding genes was discovered to encode a predicted beta-lactamase fold type protein. The second gene encoded a predicted protein resembling a group of uncharacterized isochorismatase-like proteins that could possibly be related to pyrazinamidases. These enzymes are known for their ability to degrade pyrazinamide (PZA) used in treatment against *Mycobacterium tuberculosis* infections [29].

The two encoded membrane-proteins were predicted to contain 9 and 12 transmembrane helices (TMH), respectively. The second, larger protein, showed significant homology to the large and diverse group of major-facilitator superfamily (MFS) permeases, making it likely that the two membrane proteins constituted a multi-drug efflux mechanism [30]. Although there was considerable genetic evidence pointing towards an antimicrobial resistance mechanism within this region, drug resistance beyond ampicillin and streptomycin (against which neither *Shigella* sp. MO17 or *Shigella* sp. MO440 conferred resistance) was not investigated any further in this study.

The second putative genetic load region displayed over 99% sequence homology to a section of *E. coli* ATCC 8739 which contained a predicted type 1 fimbrial biogenesis cassette. The corresponding region in pMO17/440_54 was flanked by IS1-elements, and had therefore most likely been lifted from *E. coli* by the incorporation of these elements (thus forming a composite transposon). However, the transported fragment did not contain all the necessary components for complete fimbrial biogenesis via the chaperone-usher pathway [31]. Most notably, the gene encoding the large Usher-protein homologue (FimD), had been heavily truncated, while a putative FimA component was completely lacking. The cassette still encoded a predicted surface protein (consistent with the adhesin component of type 1 fimbrial systems), and a FimC chaperone-homologue. The downstream region of the putative biofilm biogenesis genes, encode a predicted inner membrane protein with 3 TMH’s but did not show homology to known fimbrial components.

### pLN126_33 and R485

Plasmids R485 and pLN126_33 were found to contain 81 and 45 putative protein coding sequences, respectively. Compared to the highly mosaic pMO17/440_54 they were considerably simpler in structure, although, similarly, both backbone regions have been compromised by the insertion of transposable elements.

#### Plasmid maintenance regions

In both cases, the plasmids contained regions dedicated to replication via the IncX-mechanism, although the π/RepX protein in R485 diverged considerably from the pOLA52 equivalent, instead showing 100% homology to the π protein of the plasmid pMAS2027 (GenBank ID: FJ666132). Furthermore, they also harboured the associated stability determinants in the form of the *parFG* partitioning and *stbDE* stability loci, as described in the previous section.

The sequence divergence observed between the *repX* genes of pOLA52 and R485 raises the question these two plasmids should be assigned to the same IncX1 group [4]. However, we believe that this discussion, albeit important, is outside the scope of this study.

#### Horizontal transfer

Both pLN126_33 and R485 encoded regions with close resemblance to the pOLA52 *mob* region, responsible for DNA transfer and replication (Dtr) [32]. The region consists of a relaxases/nickase encoded by the *taxC* gene and an accessory protein encoded by the *taxA* gene. Furthermore, both plasmids harboured the two *oriTs* contained within long inverted repeats (LIRs), where one is located near the *taxC* gene and the other is located close to the *repX* gene. Two *oriTs* have so far been recognized as a unique characteristic of conjugative IncX-plasmids. A third gene *ddp3* (which has also been linked to the Dtr mechanism) was located near the second *oriT* in both plasmids. The plasmid R485 has previously been investigated for its ability to conjugate [33], and here it was observed that R485 conjugation on solid surfaces was massively favoured over liquid media (surface obligatory). This was in contrast to the related IncX plasmids R6K and TP228, which only displayed a *preference* towards solid surfaces (almost a thousand fold less than R485).

**Table 1:** Reported occurrences of type 3 biofilm promoting IncX-plasmids.

Plasmid	Size (kb)	Host Strain	YOI	Origin	Sample type	Reference
pOLA52	52	*Escherichia coli*	1998	Denmark	Swine Manure	11
pLN126_33	33	*Shigella* sp. LN126	2007	Denmark	Wastewater	This study
R485	61	*Morganella morganii* M203	1972	USA	UTI patient	This study
pMAS2027	43	*Escherichia coli* MS2027	2007	Australia	UTI patient	5

Unfortunately, in the only recovered Entranceposon derivative of pLN126_33, the transposons was inserted directly into the *taxC*-gene, meaning that the plasmids mobilization capabilities could not be investigated experimentally.

The cognate type IV secretion system (T4SS), or *tra* loci, which encode protein products that ensure mating pair formation (Mpf) and subsequent relaxosome transfer to the recipient, were in both plasmids the same as the pOLA52 T4SS. The large T4SS-encoding operon had however been compromised in both cases by different insertions of IS elements.

**Figure 3:**
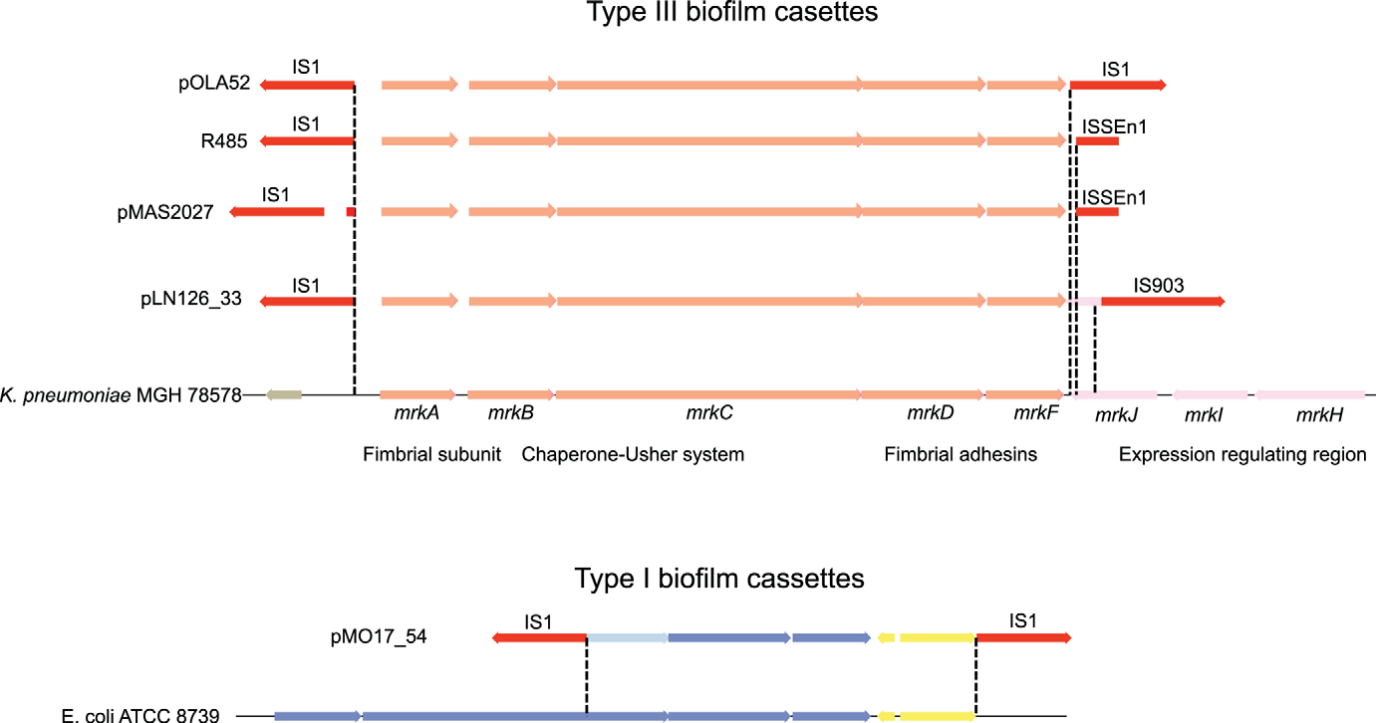
Fimbriae encoding gene cassettes. (top) The different versions of the *mrkABCDF* cassette and their flanking IS elements as they are arranged on plasmids pOLA52, R485, pMAS2027 & pLN126_33, compared to the corresponding chromosomal region of *K. pneumoniae* MGH 78578. (bottom) A similar comparison between the inserted region of plasmids pMO17/440_54 and the corresponding *E. coli* region that contains a putative type 1 fimbrial biogenesis cassette.

**Figure 4:**
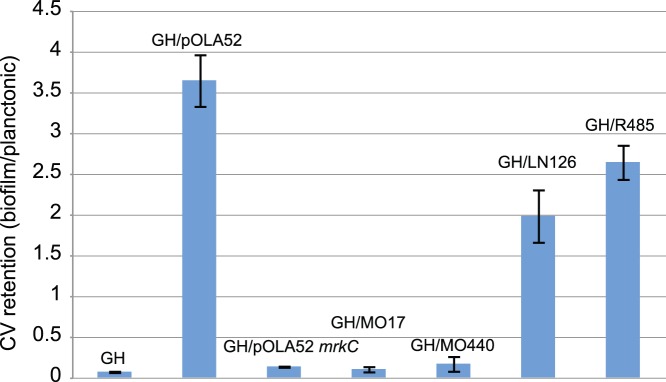
Biofilm induction by IncX-plasmids containing fimbrial gene cassettes. Biofilm formation measured via a crystal violet retention assay in *E. coli* Genehogs (GH) carrying pOLA52 compared with that of plasmids pMO17_54, pMO440_54, pLN126_33 and R485, respectively. The *mrkC* mutant of pOLA52 has a disrupted Usher protein-encoding gene and can therefore not produce biofilm. Error bars are standard deviations (*n* = 4).

#### Genetic load region

A large (36.4–51.7 kb) inserted genetic load segment, which disrupted the *topB* gene (normally encoding a topoisomerase) in R485, was apart from some *rep* and *tra* elements, found to contain a cassette encoding dihydropteroate synthase (EC 2.5.1.15) in the form of the *sulII* gene and an 80% C-terminal truncated phosphoglucosamine mutase via the *glmM* gene. Dihydrooptereorate synthase is the target for the sulfonamide group of antimicrobial agents, which block the tetrahydrofolate synthesis pathway in bacteria. The presence of the *sulII* gene on R485 is therefore in all likelihood the mechanism by which the plasmid confers sulfonamide resistance [34].

In addition, the inserted region also contained an *arsRHSB* operon that in all likelihood encodes an arsenic/arsenate resistance mechanism [35] a type of resistance not previously been described in R485.

Surprisingly, the type 3 fimbrial cassette previously characterized in both pOLA52 and the conjugative plasmid pMS2027 isolated from uropathogenic *E. coli* (UPEC) were found also to be present in both R485 and pLN126_33 (Table 1) [4], [10], [36]. In fact, the only clearly adaptive load present on pLN126_33 seems to be the *mrkABCDF* cassette. Since only the UPEC strain was specifically selected for its ability to produce biofilm, it is quite remarkable that this cassette was found on in three otherwise unrelated studies. The presence of the *mrkABCDF* cassette on the R485 plasmid also explains the previous observations made by *Bradley et al.* that R485 confers two different types of surface pili. By deduction, the two types of pili observed were the IncX-pilus (thick rigid) and the type 3 fimbriae (thin flexible), respectively [6], [33].

As was the case with pOLA52 and its Tn*6011* transposon, the cassettes were all flanked by IS-elements and displayed very high homology to the *Klebsiella pneumoniae* MGH 75878 *mrkABCDF* cassette [4]. Sequence comparison revealed two 9 bp (in-frame) deletions within the fimbrial cassette of R485, which were shared with the pMAS2027 cassette and a unique 12-bp deletion, but otherwise the sequences contained few single-nucleotide polymorphisms compared to the pOLA52 or *K. pneumoniae* MGH 75878 cassettes. The inserted fragments were not flanked by identical IS-elements at the *mrkF* end **(** **Fig. 3** **)**, possibly indicating a history of gradual shaping of the cassette through progressive invasions of new IS-elements closer and closer to the encoding genes. Interestingly, the pLN126_33 plasmid, which was isolated 35 years later than R485 [37], contained the longest of the putative *K. pneumoniae* fragments. The R485 fragment was identical to the pMAS2027 fragment with respects to the flanking IS-elements, but the both R485 and pMAS2027 contained indels not present in the other plasmid. The pLN126_33 cassette also encodes a highly truncated gene from the downstream region of *mrkF*. In *K. pneumoniae* this region contains the *mrkHIJ* control cassette [38], [39], where *mrkJ* encodes an EAL-domain phosphodiesterase shown to regulate expression of *mrkABCDF* through cleavage of the signalling molecule cyclic-di-GMP [40]. The existence of three different lengths of mobile *mrkABCDF* cassettes suggests a general course of evolution for adaptive loads that result from the recruitment of chromosomal gene cassettes. We speculate that this recruitment would start with a long genomic fragment being captured by the random incorporation of IS-elements, which subsequently find their way into the communal gene pool [1]. Over time, redundant parts of the gene cassettes are gradually lost by the constant incorporation of new IS-elements. Only those insertions that do not disrupt the adaptive phenotype of the transported cassette would be allowed to survive through natural selection.

### Biofilm Induction

The presence of type 3 fimbriae encoding cassettes on plasmids pLN126_33 and R485, and the truncated type 1 fimbriae-type cassette on pMO17/440_54, respectively, prompted a closer examination of the biofilm promoting capabilities of these plasmids. All plasmids (hosted in *E. coli* GeneHogs®) were therefore tested for their ability to enhance biofilm formation in microtitre wells, in a CV retention assay previously used to measure biofilm promotion in pOLA52 **(** **Fig. 4** **).** Both R485 and pLN126_34 displayed at least a 20-fold increase in biofilm formation compared to the plasmid-free strain and the Usher protein-disrupted *mrkC* mutant of pOLA52. Interestingly, there was a significant difference between pLN126_34 and R485, where R485 seemed capable of inducing biofilm formation more efficiently than pLN126_33. Compared to the pOLA52 plasmid, however, both the plasmids R485 and pLN126_33 promoted biofilm formation at a significantly reduced rate.

Both of the plasmids pMO440_54 and pMO17_54 were unable to enhance biofilm formation when compared to both the control strain (not carrying any plasmids) and the *mrkC* disrupted clone of pOLA52. Since the cassette in the two plasmids were not intact, when compared with the *E. coli* genome from which they had most likely been captured, this does not seem surprising. It therefore remains an open question which selective advantage the presence of this partial type 1 fimbrial cassette provide their host with, if any.

### Concluding Remarks

Plasmids belonging to the IncX group have until recently received little attention with respects to the range of adaptive genes that they are able to transport between members of the *Enterobacteriaceae*. The recent advances in sequencing technologies have now drastically improved the speed with which plasmids can be sequenced once they have been isolated enabled us to characterize several such plasmids in a single study. In order to procure a diverse range of new IncX plasmids for sequencing, a replicon-centric method of plasmid capture (TAPC) was used that allowed the isolation of naturally occurring plasmids from strains of bacteria containing high numbers of different plasmids, without prior antibiotic selection. Although the approach only led to the isolation of three IncX plasmids, of which two turned out to be identical, the method nevertheless is an effective supplement to methods such as exogenous plasmid isolation, that are dependent on a functional conjugation apparatus and specific selection pressures.

Several studies have so far reported the existence of conjugative plasmids that carry with them the ability to promote cell attachment and biofilm formation on abiotic through the formation of surface fimbriae [4], [36], [41], [42]. In the majority of cases these plasmids have been reported from within the *Enterobacteriaceae*, and within plasmids belonging to incompatibility groups (IncA, IncX) that normally thrive within this family. Comparative studies indicate that the *mrkABCDF* cassette has most likely migrated from pathogenic *K. pneumoniae,* who derive much of their virulence from their ability to form biofilm, onto several IncX plasmids either by multiple capture events, or by proliferation of a specific gene cassette in the form of a composite transposon such as Tn*6011*. The IncX plasmids therefore seem particularly capable of facilitating the spread of this cassette to strains of *E. coli,* which in effect will raise the virulence of receiving strains considerably. It is interesting to note that a specific association seems to exist between the *mrkABCDF* cassette and IncX-replicon plasmids containing whole or partial PilX-type T4SSs even though plasmids have come from vastly different sources and locations (Table 1). This either indicates that the IncX-replicon plasmids are particularly well suited to carry the *mrkABCDF* cassette, or that its presence somehow augments already-encoded functions on the IncX-backbone. Although one study pointed to a lacking ability of IncX T4SSs to promote biofilm on their own [5], there still seems be enough reason to further investigate the interplay between components of the *mrkABCDF* cassette and the PilX-type T4SSs.

A previous study of pOLA52 for example hinted that the Tn*6011* transposon dramatically enhances conjugation. Several studies have indicated that just by maintaining high conjugation frequencies a plasmid can stably propagate within bacterial communities [43]–[45]. Hence, genes such as the *mrkABCDF* cassette, that enhance conjugation frequencies indirectly by promoting biofilm formation will automatically become advantageous for the plasmid that carry them, underlining the interconnection between plasmids and biofilm formation [46].
