# Correction: Sequencing of IncX-Plasmids Suggests Ubiquity of Mobile Forms of a Biofilm-Promoting Gene Cassette Recruited from *Klebsiella pneumoniae*

**DOI:** 10.1371/annotation/b1b3cf4b-4aa1-4383-a615-d43eabdfc402

**Published:** 2012-08-09

**Authors:** Mette Burmølle, Anders Norman, Søren J. Sørensen, Lars Hestbjerg Hansen

The following information is missing from the Funding section: This study was partly funded by grants from the Lundbeck Foundation, DK nr R44-A4384 (Lars Hestbjerg Hansen), The Danish Council for Independent Research, Natural Sciences (Søren Sørensen), and The Danish Council for Independent Research, Technology and Production, ref no: 09-090701 (Mette Burmølle). 

